# Comorbid argyrophilic grain disease in an 87-year-old male with spinocerebellar ataxia type 31 with dementia: a case report

**DOI:** 10.1186/s12883-020-01723-2

**Published:** 2020-04-15

**Authors:** Shuta Toru, Shoko Ishida, Toshiki Uchihara, Katsuiku Hirokawa, Masanobu Kitagawa, Kinya Ishikawa

**Affiliations:** 1 Department of Neurology, Nitobe Memorial Nakano General Hospital, 4-59-16 Chuo, Nakano-ku, Tokyo, 164-8607 Japan; 2 Department of Pathology, Tokyo Medical and Dental University, 1-5-45 Yushima, Bunkyo-ku, Tokyo, 113-8519 Japan; 3 Department of Pathology, Nitobe Memorial Nakano General Hospital, 4-59-16 Chuo, Nakano-ku, Tokyo, 164-8607 Japan; 4 Department of Neurology, Tokyo Medical and Dental University, 1-5-45 Yushima, Bunkyo-ku, Tokyo, 113-8519 Japan

**Keywords:** Autopsy, Spinocerebellar ataxia type 31, Argyrophilic grain disease, Case report

## Abstract

**Background:**

Spinocerebellar ataxia type 31 (SCA31) is not usually associated with dementia, and autopsy in a patient with both conditions is very rare.

**Case presentation:**

An 87-year-old male patient presented with ataxia and progressive dementia. Genetic testing led to a diagnosis of SCA31. Fifteen years after his initial symptoms of hearing loss and difficulty walking, he died of aspiration pneumonia. A pathological analysis showed cerebellar degeneration consistent with SCA31 and abundant argyrophilic grains in the hippocampal formation and amygdala that could explain his dementia.

**Conclusions:**

This is the first autopsy report on comorbid argyrophilic grain disease with SCA31.

## Background

Spinocerebellar ataxia type 31 (SCA31) is an autosomal dominant pure cerebellar ataxia and is not usually associated with cognitive decline. Here we report autopsy findings from an 87-year-old Japanese male SCA31 patient with dementia. Argyrophilic grains were found to be responsible for the cognitive decline. Neuropathological examination showed findings compatible with SCA31 in the cerebellum accompanied by the finding of argyrophilic grains. This is an interesting condition of comorbid argyrophilic grain disease (AGD) with SCA31.

## Case presentation

**Fig. 1:**
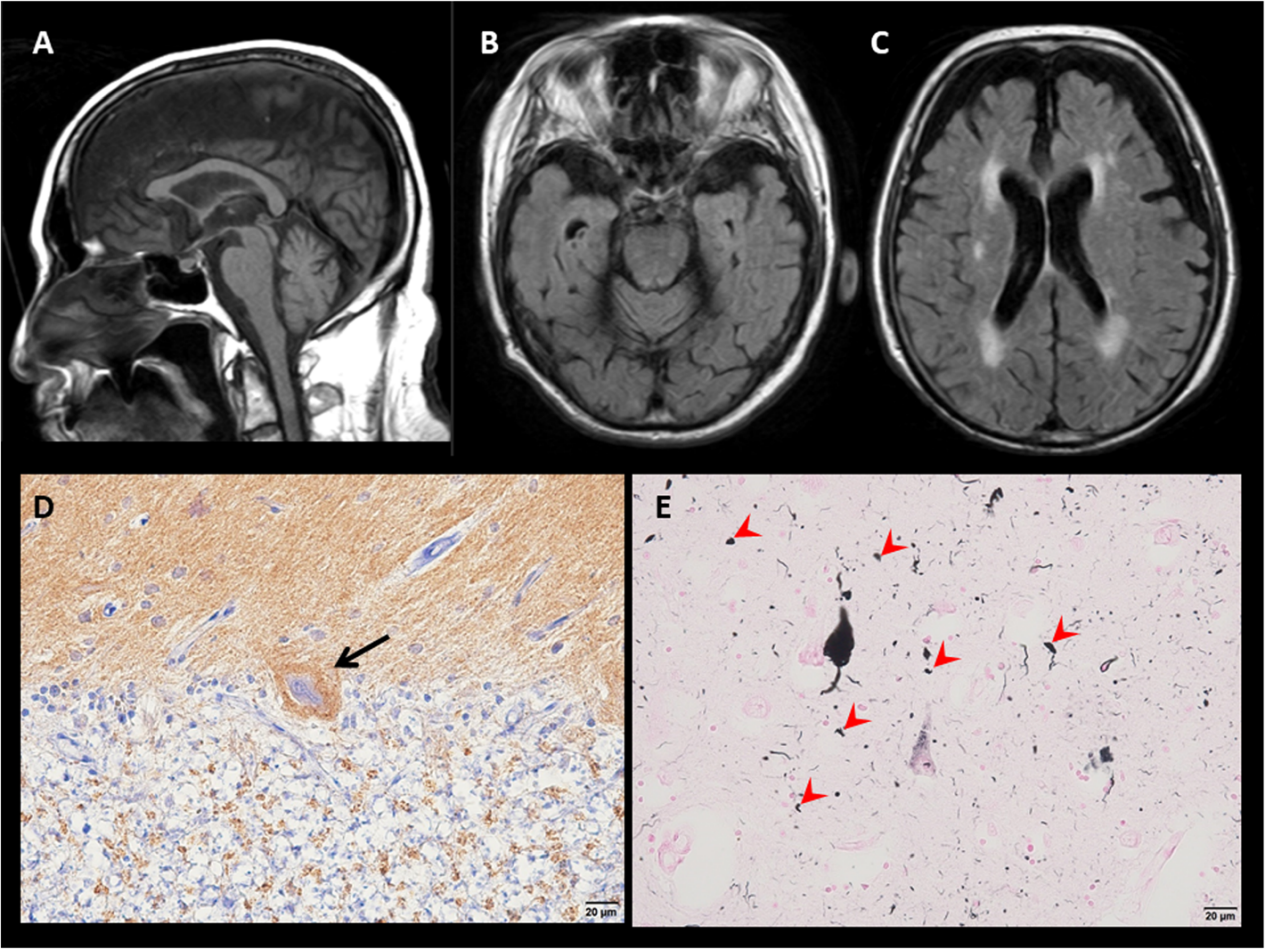
**a**: Sagittal view of a T1-weighted brain MR image showing mild atrophy of the upper vermis of the cerebellum. **b,c**: An axial MRI (fluid-attenuated inversion recovery image) showed mild atrophy in the upper vermis of the cerebellum and the right dominant anterior medial temporal lobe (**b**), and moderate atrophy of the frontotemporal cortex (**c**). **d**: Synaptophysin staining shows an eosinophilic halo-like structure (arrow) around a degenerated Purkinje cell in the right cerebellar hemisphere. **e**: Gallyas-positive, rod-like (arrowheads) or granular argyrophilic grains in the subiculum of the left hippocampus

**Fig. 2:**
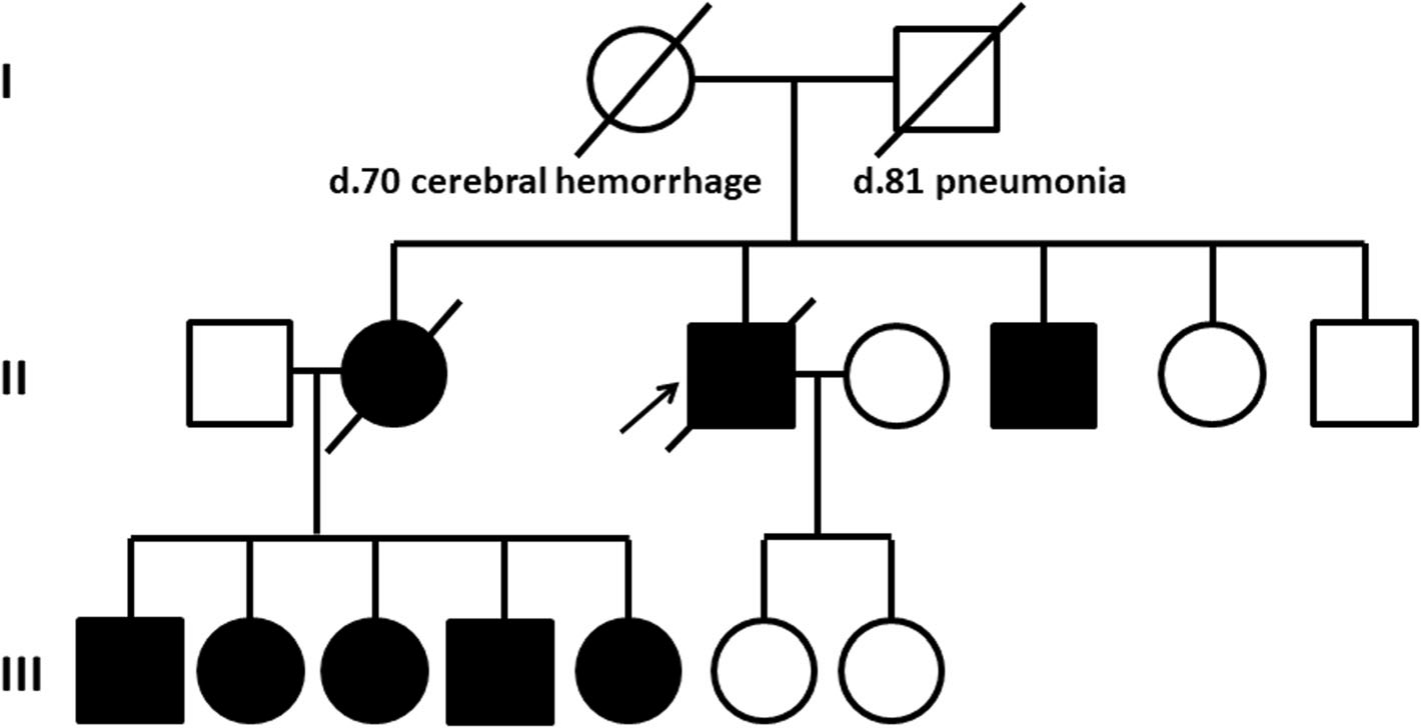
Family tree of the patient. Arrow shows the proband. Black boxes indicate family members who had cerebellar ataxia

A male patient consulted with our hospital at the age of 77. He had had hypertension since age 67. Hearing loss appeared when he was 72 years old and difficulty in walking when he was 73. Dysarthria and difficulty with hand movements had gradually appeared. At presentation, limb and truncal ataxia and ataxic speech were seen, as was hyporeflexia in the distal extremities. Brain magnetic resonance imaging (MRI) showed atrophy of the upper vermis of the cerebellum (Fig. 1a,b), as well as moderate atrophy of the frontotemporal cortex, including the right-dominant medial temporal lobe (Fig. 1b,c). Two siblings and five nephews and nieces had similar dysarthria and gait disturbances (Fig. 2). Genetic analysis revealed a TGGAA repeat expansion in the SCA31 locus, and he was diagnosed with SCA31. An audiogram showed sensorineural deafness of the downward sloping type. His score on the Mini-Mental State Examination (MMSE) was 26/30. Neuropsychological assessment with the Miyake Memory Test and Rey-Osterrieth Complex Figure Test revealed short-term memory and performance impairment. As his activity and intelligence gradually declined, he needed home-visit medical treatment. His MMSE score was 16/30 at the age of 81 and he became irritated frequently. At the age of 84, he developed a resting tremor in the right hand. He died at home with aspiration pneumonia at the age of 87, after a disease duration of 15 years.

The autopsy confirmed bilateral pneumonia as the direct cause of death. The brain weighed 1060 g. Macroscopically, the cerebellum was severely atrophic and cerebral atrophy was mild. Although the volume of the hippocampus and amygdala were relatively well preserved, white matter atrophy was accentuated in the parahippocampal gyrus. Histologically, marked neuronal loss of Purkinje cells was seen in most of the cerebellar cortex. In the remaining Purkinje cells, degeneration accompanied by cytoplasmic atrophy and nuclear aggregation was noticeable. An eosinophilic halo-like structure was present around the degenerated Purkinje cells and showed positive staining for synaptophysin (Fig. 1d). These findings are consistent with those previously reported for SCA31 [1]. Numerous Gallyas-positive, rod-like or granular argyrophilic grains were found in the ambient gyrus, amygdala, hippocampus CA1, parahippocampal gyrus, and subiculum of the hippocampus (Fig. 1e). Apparent spongiform degeneration and neuron loss with gliosis were observed in the ambient gyrus and amygdala, compatible with stage II AGD [2].

Neurofibrillary tangles were limited (Braak neurofibrillary stage II) and senile plaques were seen in the neocortex, parahippocampal gyrus, and precentral gyrus (Braak neurofibrillary stage B-C).

Very few alpha-synuclein–positive deposits were present in the dorsal nucleus of the vagus nerve, locus coeruleus, and substantia nigra.

## Discussion and conclusions

SCA31 is characteristically a pure cerebellar ataxia, but this patient presented clinically with dementia as well as progressive cerebellar ataxia. In previous reports, dementia was noted in 0% [3], 5% [4], 11.9% [5], and 58% [6] of patients with SCA31.

The only reported autopsy of a patient with SCA31 and dementia [7] showed moderate tau and amyloid pathology in the neocortex that might have led to dementia at the terminal stage. However, in our case, neither the amyloid deposits nor the neurofibrillary pathology was sufficient to explain the patient’s dementia. Instead, abundant argyrophilic grains may better explain the cognitive decline in this case. The asymmetric atrophy of the anterior medial temporal lobes seen with MRI is a diagnostic feature of AGD and is consistent with the diagnosis in this case. SCA31 usually has an older age at onset than other autosomal dominant hereditary ataxias, which provides more chance to develop age-related cognitive decline such as Alzheimer disease. However, our case shows that it is necessary to consider argyrophilic grains as a pathological substrate for cognitive decline in patients with SCA31 even though the coexistence of SCA31 and AGD is extremely rare.

This is the first autopsy report on comorbid AGD with SCA31, providing a clue for neuroradiological diagnosis of grain disease.

## Data Availability

Not applicable.

## Abbreviations

SCA31: Spinocerebellar ataxia type 31
MMSE: Mini-Mental State Examination
AGD: Argyrophilic grain disease
MRI: Magnetic resonance imaging
